# The etiology of Bell’s palsy: a review

**DOI:** 10.1007/s00415-019-09282-4

**Published:** 2019-03-28

**Authors:** Wenjuan Zhang, Lei Xu, Tingting Luo, Feng Wu, Bin Zhao, Xianqi Li

**Affiliations:** 1 Shanxi Medical University School and Hospital of Stomatology, Taiyuan, 030001 China; 2 The State Key Laboratory Breeding Base of Basic Science of Stomatology and Key Laboratory for Oral Biomedical Engineering of Ministry of Education, School and Hospital of Stomatology, Wuhan University, Wuhan, China; 3 Department of Oral and Maxillofacial Surgery, School of Dentistry, Matsumoto Dental University, 1780 Hirooka Gobara, Shiojiri, 399-0781 Japan

**Keywords:** Bell’s palsy, Cold stimulation, Viral infection, Inflammation, Anatomical structure, Ischemia

## Abstract

Bell’s palsy is the most common condition involving a rapid and unilateral onset of peripheral paresis/paralysis of the seventh cranial nerve. It affects 11.5–53.3 per 100,000 individuals a year across different populations. Bell’s palsy is a health issue causing concern and has an extremely negative effect on both patients and their families. Therefore, diagnosis and prompt cause determination are key for early treatment. However, the etiology of Bell’s palsy is unclear, and this affects its treatment. Thus, it is critical to determine the causes of Bell’s palsy so that targeted treatment approaches can be developed and employed. This article reviews the literature on the diagnosis of Bell’s palsy and examines possible etiologies of the disorder. It also suggests that the diagnosis of idiopathic facial palsy is based on exclusion and is most often made based on five factors including anatomical structure, viral infection, ischemia, inflammation, and cold stimulation responsivity.

## Background

Bell’s palsy (BP), named after the Scottish anatomist Sir Charles Bell, is the most frequent diagnosis linked to facial nerve palsy/paralysis as well as the most frequent acute mono-neuropathy. It affects individuals across multiple ages and both sexes, with an annual incidence ranging from 11.5 to 53.3 per 100,000 persons across multiple populations [1–4]. Typically, BP results in partial or complete inability to automatically move the affected side of the facial muscles. Although it usually resolves within weeks or months, BP facial paresis/paralysis may lead to severe temporary oral insufficiency and an incapability to close the eyelids in some cases, resulting in potentially permanent eye injury. In approximately 25% of patients with BP, moderate-to-severe facial asymmetry may persist, frequently impairing patients’ quality of life [5]. These are among BP’s long-term adverse consequences, which can be devastating for patients.

Despite its severe effects, the exact etiology of BP remains unclear. The Guideline Development Group (GDG) [6] has identified the diagnosis of BP as one of exclusion, requiring careful clinical elimination of other potential etiologies of facial paralysis/paresis, such as trauma, neoplasms, congenital or syndromic problems, postsurgical facial paralysis/paresis, or infection by agents including zoster and Lyme disease. This diagnosis also fails to address cases of recurrent facial paresis/paralysis. The GDG has also recognized the “acute” or “rapid onset” nature of BP and that the occurrence of paralysis /paresis usually reaches its maximum severity in less than 72 h of paralysis/paresis onset. But the current literature similarly lacks a precise etiology for the acute onset of facial palsy.

In this review, we sought to summarize potential clinical etiologies of BP, through search for eligible studies on PubMed, Embase, and the Web of Science up to 31 October 2018 using the following search terms: acute facial paresis/paralysis, Bell’s palsy, idiopathic facial palsy, and/or etiology. There are five major theories regarding the causes of BP including anatomical, viral infection, ischemia, inflammation, and cold stimulation (Tables 1, 2, 3, 4, 5).

**Table 1:** Summary of key evidence for the etiological theory about anatomical structure

Key references	Summary of evidence
[9]	Yilmaz et al. detected lower internal auditory canal (IAC) inlet as well as mid-canal values in the patients with Bell’s palsy
[10–12]	The cross-sectional areas (CSAs) of facial nerve (FN) were larger and the CSAs of IAC were smaller on the influenced sides than the equivalents on the uninfluenced sides of the patients, respectively. There is a significant difference between influenced and uninfluenced sides of the patients in terms of the mean CSA of the FN and IAC (*p* < 0.001). Bell’s palsy seems to usually coincide with the narrower fallopian tube of the patient
[13, 14]	The mean width was significantly smaller at the labyrinthine section of the facial canal in the influenced temporal bone than the equivalent in the uninfluenced (*p* = 0.00)
[14]	Significant relationship was found between the HB grade and the facial canal diameter at the level of second genu (*p* = 0.02)
[9]	In patients with higher primary HB-scores, their 6-month later HB-scores were also higher. In patients with higher 6-month HB score; their IAC inlet and mid-canal values were lower

**Table 2:** Summary of key evidence for the etiological theory about virus infection

Key references	Summary of evidence
[21–24]	The α-HV which target peripheral neurons (e.g., HSV-1, HSV-2, and VZV) can establish lifelong infections and infectivity potential in the host including in the autonomic and sensory ganglia of the head, neck and cranial
[25]	Reactivation of HSV-1 centered around the geniculate ganglion was first outlined by McCormick in 1972
[26]	The presence of HSV-1 deoxyribonucleic acid (DNA) was detected in clinical specimens, i.e., intra-temporal facial nerve endo-neural fluid in Bell’s palsy patients
[27–29]	Animal models have the capability to cause facial paralysis through initial infection and virus reactivation incited by immune modulation
[36, 37]	Earlier work examining cellular electrophysiology in the setting of herpes infection demonstrated a pathway for the quick and dynamic control of excitability in sensory neurons by internalization of sodium channels. The processes of intra-axonal degeneration would drive the abrupt onset of Bell’s palsy
[38]	The aquaporin 1 water channel protein (AQP1) in Schwann cells of intratemporal facial nerve is involved in the evolution of facial palsy caused by HSV-1 and may play an important role in the pathogenesis of this disease
[39]	Decreasing LAT levels in neurons reduced the ability of the virus to reactivate. This suggests the potential of reverse validation of bell’s palsy as a virus reactivation

**Table 3:** Summary of key evidence for the etiological theory about ischemia

Key references	Summary of evidence
[46]	Endoneurial blood supply to peripheral nerves is not uneven. And endoneurial capillary density corresponds to the level of sensitivity to ischemic nerve damage in experimental and human ischemic neuropathies
[48, 49]	It is possible certainly, as is witnessed by the onset of acute facial palsy following the embolization of cerebral venous or dural arteriovenous fistula
[50]	To establish an animal model of ischemic facial nerve palsy in rats, observe the internal vascular network of facial nerve in fallopian canal, the facial nerve palsy appears within 5–15 min after selective arterial embolization, and the internal capillaries of the facial nerve appeared to be thinner, some of which are blocked by microspheres, especially in the labyrinthine segment
[52]	After removing the bony covering, researchers observed the facial nerve swelling in patients with facial paralysis, and they found nerves dilation in diameter by 12–32% (mean 21.0 ± 6.1%). Injection and exudate were also observed among these patients
[43, 55]	Among those cases that cannot recover, the facial nerve sheath become thick, forming one or more fibrous bands that cause nerve strangulation and compression, thereby hampers its recovery

**Table 4:** Summary of key evidence for the etiological theory about immune inflammatory

Key references	Summary of evidence
[57]	Histologic changes in the facial nerve, found by Liston and Kleid, that can be summarized as follows:(1) the nerve, from the internal acoustic meatus to the stylomastoid foramen, is infiltrated by round, small inflammatory cells. (2) There was A breakdown of neuron myelin sheaths, which involved macrophages, occurs. (3) Inter-neuronal space increased. (4) The bony fallopian canal is normal, with no sign of facial nerve compression by the fallopian canal bone
[60–63]	In recent years, it is found that the mean neutrophil-to-lymphocyte ratio and neutrophil values were higher in adult and pediatric patients with Bell’s palsy
[64, 65]	Similar changes in peripheral blood leukocyte subpopulations are also described in the process of a few inflammatory demyelinating diseases, such as during the acute stage of Guillain–Barré syndrome and in acute exacerbations of multiple sclerosis. Bell’s palsy, such as Guillain–Barré syndrome, may be an acute demyelinating disease of the peripheral nerve system
[68]	An examination of the serum samples from patients with Bell’s palsy showed elevated concentrations of cytokines interleukin-6 (IL-6), interleukin-1 (IL-1), and tumor necrosis factor-alpha (TNF-α) were increased compared with control groups
[69, 70]	In contrast to control populations, decreased percentages of total T cells (CD3) and T helper/inducing cells (CD4) have also been found in the acute phase of the disease. An obviously decreased peripheral blood T lymphocyte percentages and an increase in B lymphocyte percentage in BP have been found within the first 24 days from the clinical onset of the paralysis. These evidences indicate an activation of cell-mediated effectors and the involvement of immune mechanisms in Bell’s palsy

**Table 5:** Summary of key evidence for the etiological theory about acute cold exposure

Key references	Summary of evidence
[77]	Some authors estimated rates and trends of Bell’s palsy using a centralized surveillance system. They found both season and climate (adjusted ratio of cold to warm months = 1.31) were independent predictors of risk of Bell’s palsy
[78–80]	There is a clear relationship between the cold season and the number of cases observed. However, some researchers have found that BP is more frequent in warm seasons (spring and summer), with its incidence peaking in September
[81]	More deeply, one study evaluated the influences of meteorological factors on the incidence and onset of BP. Evidence suggests that stronger wind speed of preceding day may be related to the occurrence of Bell’s palsy
[82]	One study retrospectively reviewed 568 files of Bell’s palsy patients and concomitant data of meteorological factors. The result showed the number of cases per month was significantly and negatively was significantly and negatively correlated with the summer months and mean monthly temperatures (*p* = 0.002 and < 0.000, respectively) and strong positive correlation with monthly wind chill factor (*p* < 0.000). Wind chill factor is a novel, reliable estimator of the overall meteorological factors-derived risk
[83]	A community-based research in Qena Governorate, Egypt confirmed the most frequent precipitating factors for an episode of Bell’s palsy were exposure to air draft in 40%. This could be related to variations between day and night temperatures in their community. Sharp temperature changes may be one of the risk factors for facial nerve palsy, and especially the susceptibility to air draft exposure during the night

## Anatomical structure

The facial nerve (CN VII) is a unique motor nerve, emerging from the facial nerve nucleus in the pons. From this point, it is accompanied by the CN VIII along its cisternal pathway to the internal auditory meatus. Specifically, its petrous route includes a labyrinthine segment, a horizontal tympanic segment, and a vertical mastoid segment, which extends until it reaches the stylomastoid foramen and the parotid gland. CN VII then follows a long-drawn-out pathway through the temporal bone within the fallopian canal [7, 8]. Given this extended and convoluted pathway, it is more susceptible to palsy than other nerves in the body.

Anatomical differences are critical in assessing cases of BP. After Yilmaz et al. [9] assessed the lower internal auditory canal (IAC) inlet as well as mid-canal values in patients with BP, Ozan and Arslan [10] compared the diameters and cross-sectional areas (CSAs) of the facial nerve (FN) and IAC between the affected and unaffected sides of 56 patients via three-dimensional fast imaging, employing steady-state acquisition magnetic resonance imaging (MRI). They also found a notable difference between the affected and unaffected sides of the patients in terms of their mean CSAs of the FN and IAC (*p* < 0.001). The CSAs of the FN were larger, and the CSAs of the IAC were smaller on the affected side than their counterparts on the unaffected side. Differences in the ratios of IAC CSA to FN CSA between the affected and unaffected sides of the patients were also found to be statistically significant (*p* < 0.001). Collectively, these data suggest that BP generally coincides with a narrower fallopian tube in affected patients. These anatomical differences, supported by previous MRI studies, may thus contribute to disease risk [11, 12].

In their more detailed study, Celik et al. [13] found that the facial canal’s mean width at the labyrinthine section in the affected temporal bone was much smaller than the respective one in the unaffected side (*p* = 0.00). In that retrospective clinical study, the authors used temporal computed tomography and found no notable differences between the affected and unaffected temporal bones at the geniculate ganglion, second genu, tympanic segment, mastoid segment, and the stylomastoid foramen except for the labyrinthine segment. Therefore, the facial canal’s diameter of the labyrinthine segment is possibly an anatomical risk factor of BP. Celik et al.’s study was consistent with the findings described by Murai et al. [14], who assessed the relation between the facial canal’s diameter and the BP grade, as evaluated with the House–Brackmann (HB) grading system. The authors did not find any relation between the HB grade and facial canal diameter at the level of the geniculate ganglion, labyrinthine segment, tympanic segment, mastoid segment, or the stylomastoid foramen, although they found a significant relation at the level of the second genu (*p* = 0.02).

With the development of 3-D imaging, future studies (3-D modeling alone or with MRI-CT) are needed to promote the potential relevance of BP to neurological disruptions, especially at the second genu. Furthermore, no significant correlations have been revealed between the paralyzed side, the primary HB stage, and IAC measurements in BP patients [9]. In patients with higher primary HB-scores, their HB-scores 6 months later had remained higher and their IAC inlet and mid-canal values were lower [9].

## Viral infection

Another possible etiology of BP that has been suggested is infection by reactivated viruses, such as the varicella zoster virus (VZV) [15], herpes simplex virus type 1 (HSV-1) [16], human herpes virus 6 [17], and the Usutu virus [18]. Herpesviruses (HV) are large, enveloped viruses with double-stranded linear DNA. α-HV infections, which target peripheral neurons (e.g., HSV-1, HSV-2, and VZV), are among the most common viral infections worldwide [19]. HSV and VZV infections can persist across the host’s lifespan [20]. α-HVs enter the human body via the mucosa and establish their latent presence in multiple ganglia of the neuroaxis by highly restricted gene transcription for the host’s entire life, including in the autonomic and sensory ganglia of the head, neck, and cranium [21–24]. When there is no active viral replication or assembly, this latent form in the ganglia is characteristic and widely distributed throughout diseased and normal populations. Both HSV and VZV can reactivate in the presence of circulating antibodies or in an immunocompetent host, while reactivation is more likely in cases of immunodeficiency.

HSV-1 is one of the most well-studied viruses. The reactivation of HSV-1 centered around the geniculate ganglion, and thus potentially linked to BP, was first outlined by McCormick [25]. An association with HSV-1 is supported by the presence of HSV-1 DNA in clinical specimens (i.e., intra-temporal facial nerve endo-neural fluid) [26] in BP patients, as well as its capability to cause facial paralysis in animal models after initial infection [27] and reactivation incited by immune modulation [28, 29]. A possible cause of HSV-1-mediated neural dysfunction is the activation of apoptotic pathways and intra-axonal degradation, which are driven by the axon’s local indirect and direct responses to the viruses themselves in susceptible phenotypes. Studies have suggested that viruses use abnormal expressions of p53 upregulated modulator of apoptosis (PUMA) [30] and/or innate immunity signaling molecule (SARM1) [31, 32] to trigger axon degeneration. This implies that PUMA and/or SARM may play an important role in HSV-1-mediated neural dysfunction.

Recent in vitro studies have demonstrated local messenger RNA transcription in peripheral nerve axons incited by α-herpes virus particles [33, 34]. In these compartmentalized models, protein and signal transduction changes are not reliant on nucleus machinery, but rather when viruses enter the axons, the axons respond locally [35]. Earlier work examining cellular electrophysiology in the setting of herpes infection demonstrated a pathway mediating the rapid and dynamic control of excitability in sensory neurons via the internalization of sodium channels [36]. The activity of sodium channels and reversal of the sodium-calcium exchanger can thus contribute to the axonal degeneration of peripheral axons after mitochondrial dysfunction and consequentially impaired activity of Na/K-ATPase [37]. These processes of intra-axonal degeneration drive the sudden onset of BP and also explain the lack of a pronounced immune response in these models. In mice with facial palsy caused by HSV-1, AQP1 protein levels increased significantly from day 9 to day 16 after inoculation of HSV-1. The upregulation of AQP1 is closely related to intratemporal facial nerve edema in the facial nerve canal, and is also consistent with the symptoms of facial palsy in mice [38]. This may in the first place, answer the question of why the nerve swells leading to impingement. In a hypoxia model of Schwann cells in vitro, the ERK antagonist U0126 not only inhibited the upregulation of phosphorylated ERK and AQP1, but also inhibited the morphological changes of Schwann cells. Combined with the upregulation of phosphorylated ERK in mice with facial palsy induced by HSV-1, it could be speculated that the ERK MAPK pathway might also be involved in the increase of AQP1 in the BP mouse model [38].

The details of how HSV reactivates are largely unknown. Most of its genes are silent during the latency period, with the exception of RNAs transcribed from the latency-associated transcript (LAT) region of the gene. After the establishment of HSV-1 latency, an adeno-associated virus delivered a LAT-targeting hammerhead ribozyme to rabbit neurons infected with HSV-1. Using this model, decreasing LAT levels in neurons reduced the ability of the virus to reactivate [39]. This suggests the potential for reverse validation of BP etiology with viral reactivation, as well as a potential avenue for the treatment of HSV infection.

Although HSV-1 has been held responsible as the most specific and major factor in BP, the behavior of patients with BP is unusual compared to that of patients with other diseases more commonly associated with HSV, such as herpes labialis (cold sores) or herpetic gingivostomatitis [40], and some BP patients do not have detectable levels of viral infection [41, 42]. Even if it is supposed that herpesviruses cause BP and there is CNS involvement in at least some patients, studies on the cerebrospinal fluid (CSF) of BP patients have suggested that it could not determine the timing and duration of viral presence in the CSF [42]. Namely, clinical evidence of HSV-1 infection in the geniculate ganglion remains elusive.

## Ischemia

BP, an acute idiopathic lower motor neuron palsy, is commonly a unilateral and self-limiting inflammatory condition. Most cases of BP remit within 4–6 months and almost always resolve completely within 1 year. Among those cases that do not resolve, studies have implicated secondary ischemia, tertiary ischemia, or their sequelae, and this in turn, can result in thickening of the facial nerve sheath, forming one or more fibrous bands that cause nerve strangulation and compression, thereby hampering recovery [43].

The nerves is a layered structure with a shiny tough grey outer periosteal layer and another layer immediately below this one that covers the epineural layer of nerve tissue and is formed by a vascular plexus. Peripherally, this vascular plexus is formed by the stylomastoid artery and is replaced by the petrosal branch of the middle meningeal artery, internal auditory artery, and the anterior inferior cerebral artery more centrally [44, 45]. A previous study showed that endoneurial capillary density corresponded to the level of susceptibility to ischemic nerve damage [46]. That is, peripheral nerve susceptibility to ischemia is at least partially determined by the density of the endoneurial capillaries. The presence of this vascular layer is clearly important, as when it is compromised, local ischemia and palsy may result. In general, vascular ischemia assumes one of three types, each outlined in a section below.

### Primary ischemia

The facial nerve has a tough epineurium with abundant vascular supply. However, vasospasms result in decreased blood supply and acute inflammation, leading to primary ischemic neuropathy, which is rare. In certain clinical conditions, such as diabetes mellitus, primary ischemic neuropathy is likely to occur. After transient ischemia and reperfusion, acute inflammation of the diabetic nerve is seen. Resident macrophages are activated and recruited for macrophage infiltration [47]. The onset of acute facial palsy after the embolization of cerebral venous [48] or dural arteriovenous fistulas [49] provides some evidence of this. An animal model of ischemic facial nerve paralysis in rats has blockages of the internal vascular network of the facial nerve in the fallopian canal. Facial nerve paralysis appears within 5–15 min of selective arterial embolization in rat models, with the internal capillaries of the facial nerve thinning and some, especially in the labyrinthine segment, becoming blocked by microspheres [50]. This evidence suggests that lack of significant anastomoses between the petrosal branches and stylomastoid may predispose individuals to ischemia.

### Secondary ischemia

Hilger found that the process of primary ischemia could result to secondary ischemia [51]. The basic characteristics of secondary ischemia in these cases involve initial constriction of the arterioles, followed by capillary dilatation, which in turn results in permeability increases and consequent exudation. The lymphatic capillaries are then compressed by the transudate, with some even closing. This further increases the formation of exudate and causes regional ischemia, producing a vicious cycle that may even lead to nerve necrosis and ultimately discontinuity of the nerve.

Clinical evidence has suggested some links between secondary ischemia and BP. Hagino et al. [52] reported that in patients with facial palsy that underwent facial nerve decompression, there was facial nerve swelling, injection, and exudate, as well as nerve diameter dilation by 12–32%. The underlying pathophysiology observed in post-mortem cases of BP is inflammation-based, with vascular distension and edema with ischemia of the facial nerve ultimately resulting [53]. Grewal also reported that the pathophysiology of edema within the fallopian canal occurs via similar mechanisms [43]. Initial vasospastic changes increase capillary permeability and edema, supported and aided by histamine release and an intermediate hypersensitivity reaction. Any slight nerve swelling can thus lead to obstruction of venous effluents, which in turn promote poor blood circulation and interfere with the nerve’s arterial blood supply.

### Tertiary ischemia

Grewal contested that, in some cases, secondary ischemia progression leads to or progresses to tertiary ischemia [54]. This is the result of the vasospasm progressing, which can cause perivasculitis and endarteritis. These, in turn, lead to varying degrees of facial nerve sheath fibrosis, thickening of the facial nerve sheath, and sometimes to the formation of fibrous tissue, resulting in facial nerve strangulation. At this stage, some surgeons suggest surgical decompression by sheath incision, and removal of any fibrous bands is important, otherwise permanent facial paralysis can result [55]. Despite reports that patients with severe BP could benefit from decompression surgery, it is noteworthy that the role of surgical intervention in the management of BP is still disputed [56].

## Inflammation

Numerous lines of evidence have suggested that BP results from acute, inflammation-caused demyelination. The first line of evidence supporting this etiology is supported by histologic changes in the facial nerve, first identified by Liston and Kleid [57], the characteristics of which are summarized as follows. (1) The nerve, from the internal acoustic meatus to the stylomastoid foramen, is infiltrated by round, small inflammatory cells. (2) A breakdown of neuron myelin sheaths, which involves macrophages, occurs. (3) Inter-neuronal space is increased. (4) The bony fallopian canal is normal, with no sign of facial nerve compression by the fallopian canal bone.

A high neutrophil-to-lymphocyte ratio (NLR) is considered as a reliable etiological indicator of disease severity in inflammatory disorders [58, 59]. Studies have shown that the mean NLR and neutrophil values in adult and pediatric patients with BP were found to be significantly higher than in healthy controls [60–63]. This suggests changes in peripheral blood leukocyte subpopulations akin to those that occur in the process of a few inflammatory demyelinating diseases, such as during the acute stage of Guillain–Barré syndrome and during acute exacerbations of multiple sclerosis [64, 65]. Inflammatory demyelinating neuritis has been described in both diseases. The myelin sheath is a layer that wraps the axons of nerve cells and is mainly composed of Schwann cells. Schwann cells insulate axons protruding from neurons in the peripheral nervous system, thus preventing the transmission of electrical impulses from one neuron’s axon to another’s [66]. BP, as Guillain–Barré syndrome, may be an acute demyelinating disease of the peripheral nervous system. As most cases of uncomplicated BP result in full recovery, the main lesion may comprise the surrounding myelin sheaths and their native cells, the Schwann cells, but not the nerve itself. Demyelination of the facial nerve occurs with autoimmunity, which has been further supported by a recent Greco et al. review [67].

It has also been suggested that BP is actually a polyneuropathy, with facial paralysis often involving other cranial nerves. The presence of small round lymphatic cells and the breakdown of myelin sheaths are common histologic features of autoimmune responses, with viral infection prompting an autoimmune reaction against a component of peripheral myelin and resulting in the cranial nerve’s demyelination and especially facial nerve demyelination. The mechanism underlying this remains unclear. However, serum samples from patients with BP contain elevated concentrations of cytokines, including interleukin-1 (IL-1), IL-6, and tumor necrosis factor-alpha (TNF-α) compared to control group levels [68]. In contrast to control populations, decreased percentages of total T cells (CD3) and T helper/inducing cells (CD4) have also been found in the acute phase of BP [69]. Obviously decreased peripheral blood T lymphocyte percentages and increased B lymphocyte percentages in BP have been found within the first 24 days of clinical paralysis onset [70]. All the clinical and immunological data outlined above suggest activation of cell-mediated effectors and the involvement of immune mechanisms in BP.

## Acute cold exposure

There are several possible risk factors for BP, including severe preeclampsia [71], psychological factors [72], glucose metabolism abnormalities [73], radiation exposure [74], hypertension [75], and migraine [76]. Recently, epidemiological studies have revealed that the incidence of BP is also related to extreme temperature exposure. Campbell and Brundage [77] investigated climate and season correlates of BP risk using a centralized surveillance system containing medical encounter and demographic data. The results revealed that both season and climate were independent predictors of BP risk. There is a clear correlation between the cold season and the number of cases observed [78, 79]. A previous study also found that BP occurs more frequently during the spring and summer, with its incidence peaking in September and a significantly higher incidence of BP in the summer [80].

Another study evaluated the effects of meteorological factors on the incidence and onset of BP [81]. In that study, the weather conditions of 1–7 days prior to the onset of BP were included by retrospective chart review, and any possible delayed effects of meteorological factors on the onset of BP were assessed. It was found that stronger wind speeds on the day preceding BP onset might be related to its occurrence. In addition, another study retrospectively reviewed 568 files of BP patients and concomitant data of meteorological factors during an 84-month observation period. Information collected included the number of cases per month, monthly temperatures, wind speeds, and monthly wind chill factor levels. The results showed that the number of cases per month was significantly and negatively correlated with the summer months and mean monthly temperatures (*p* = 0.002 and < 0.000, respectively) and had a significant positive correlation with the monthly wind chill factor (*p* < 0.000) [82]. These results suggest that the wind chill factor, which depends on both temperature and wind speed, is a novel, reliable estimator of the overall meteorological factor derived risks that influence the probability of BP occurrence. Furthermore, a community-based study was conducted to assess the factors that precipitated cases of BP in Qena Governorate, Egypt [83]. The most frequent precipitating factors were exposure to air draft (40%) and upper respiratory tract infection (13.3%). This might be related to variations between daytime and nighttime temperatures in the community, and especially susceptibility to air draft exposure during the night. Collectively, these studies and those discussed above demonstrate that the incidence of BP increases with acute cold exposure and in places with large diurnal temperature differences, indicating that sharp temperature changes may be a risk factor for facial nerve palsy.

Subcutaneous fat serves as a defense against cold stimulation. At present, adipose tissue is considered an endocrine organ that releases various bioactive substances, called secretory factors or adipokines [84, 85]. It has also recently been proposed that adipose tissue is a member of the diffuse neuroendocrine system [86]. Under cold stimulation, the factors secreted by fat cells change, with inflammatory factors such as McP-1 and CD68 being upregulated and brain derived neurotrophic factor and neuronatin being downregulated [87, 88]. Subcutaneous fat may thus serve as a line of defense against cold stimulation, which causes the release of various secretory factors or adipokines that influence the immune system and the susceptibility to proinflammatory events such as BP [89, 90]. Whether changes in fat affect inflammatory responses, the nervous system, and acute demyelination are thus worthy of further mechanistic study.

## Discussion

Facial expression is essential to an individual’s sense of wellbeing and ability to integrate into social networks [91]. Given this, the psychological burden of facial palsy patients could be tremendous. With marked facial asymmetry and diminished facial movement, patients with facial paresis/paralysis can experience deep social distress, social alienation, impaired interpersonal relationships, and depression [92–94], which can in turn result in decreased productivity and higher health care expenses. On the treatment of BP, adding antiviral drugs to steroids may increase the probability of recovery but, if so, only to a very modest extent [95], and the use of decompression of the facial nerve for BP remains relatively controversial [96, 97].

Up to date, most BP cases have been detected without determining a definite cause. The only broadly authenticated findings are inflammation and edema of the facial nerve leading to entrapment within the facial canal, as have been detailed above. As BP is a clinical syndrome, it is completely possible that more than one disease entity produces the idiopathic facial palsies. For instance, Lyme disease is associated with facial palsy, and may be misdiagnosed as BP [98]. It is thus important to continue to identify the multiple possible causes of BP to pursue more targeted treatments.

Moreover, according to the analysis of BP onset in clinical patients, we believe that the effects of sharp temperature changes in the surrounding environment cannot be ignored. Especially when the head and face was long exposed to an extremely cold or hot temperature environment, the sharp temperature changes will easily lead to the microenvironment change of the facial microvascular neuron, which may be a high-risk factor of BP. In summary, we hope that the present review may be useful in determining the various etiologies and subtypes of BP, including cold stimulation responsivity, viral infection, and others, and thus serve as a convenient guide in the future for scholars and clinicians. It is likely that resultant treatments can thus be predicated on the specific etiologies of individual cases, thus improving outcomes and producing better cures.

## Data Availability

Drs. Li and Zhang had full access to all the data in this study and take responsibility for the integrity of the data and the accuracy of the data analysis.
